# Enhanced sensitivity to drugs of abuse and palatable foods following maternal overnutrition

**DOI:** 10.1038/tp.2016.176

**Published:** 2016-10-04

**Authors:** D Peleg-Raibstein, G Sarker, K Litwan, S D Krämer, S M Ametamey, R Schibli, C Wolfrum

**Affiliations:** 1 Department of Health Science and Technology, Laboratory of Translational Nutrition Biology, ETH Zurich, Schwerzenbach, Switzerland; 2 Center for Radiopharmaceutical Sciences, ETH Zurich, Zurich, Switzerland

## Abstract

Epidemiological studies have shown an association between maternal overnutrition and increased risk of the progeny for the development of obesity as well as psychiatric disorders. Animal studies have shown results regarding maternal high-fat diet (HFD) and a greater risk of the offspring to develop obesity. However, it still remains unknown whether maternal HFD can program the central reward system in such a way that it will imprint long-term changes that will predispose the offspring to addictive-like behaviors that may lead to obesity. We exposed female dams to either laboratory chow or HFD for a period of 9 weeks: 3 weeks before conception, during gestation and lactation. Offspring born to either control or HFD-exposed dams were examined in behavioral, neurochemical, neuroanatomical, metabolic and positron emission tomography (PET) scan tests. Our results demonstrate that HFD offspring compared with controls consume more alcohol, exhibit increased sensitivity to amphetamine and show greater conditioned place preference to cocaine. In addition, maternal HFD leads to increased preference to sucrose as well as to HFD while leaving the general feeding behavior intact. The hedonic behavioral alterations are accompanied by reduction of striatal dopamine and by increased dopamine 2 receptors in the same brain region as evaluated by post-mortem neurochemical, immunohistochemical as well as PET analyses. Taken together, our data suggest that maternal overnutrition predisposes the offspring to develop hedonic-like behaviors to both drugs of abuse as well as palatable foods and that these types of behaviors may share common neuronal underlying mechanisms that can lead to obesity.

## Introduction

An estimated 271 million people worldwide are afflicted with disorders associated with drug abuse^1^ and an estimated 1.4 billion adults are overweight, of which 500 million are classified as obese.^2^ This places a huge burden on healthcare and severely reduces life quality of afflicted individuals. Successful treatments to date are rare and, although few educational programs have been shown to be successful, there is still a high remission rate.^3^ Recently, studies have suggested common underlying uncontrolled eating habits and compulsive behavior linked to drug of abuse.^4, 5^ This is plausible as energy-rich food is intrinsically rewarding and thereby triggers hedonic feeding and could explain why some individuals continue to consume food, although their homeostatic energy needs have been met.^6^

Two powerful circuits in the central nervous system regulate feeding behavior: the homeostatic and the hedonic systems. Deregulation of either can lead to overeating, accumulation of fat stores and ultimately obesity.^6^ Under steady-state conditions, energy intake is normally metabolized to maintain metabolic rate, energy expenditure and thermogenesis to control food intake and energy expenditure and is referred to as homeostatic regulation (mediated by hypothalamic circuits) of adiposity and body weight.^7^ The hedonic circuit, often called the natural reward circuit, in contrast regulates the motivation to accomplish something desirable, such as seeking or consuming rewarding stimuli such as illegal drugs or palatable foods.^6, 8^ The core component of hedonic drive is the mesocorticolimbic dopamine pathway that involves dopamine neurons in the ventral tegmental area (VTA) and their target areas, the nucleus accumbens (NAc) and the medial prefrontal cortex. Although there is ample evidence that there is overlap in the brain regions affected by palatable foods and drugs of abuse,^5^ there is ongoing debate about the idea that food can be 'addictive' in the same sense as drugs of abuse.^5, 9^

The causes of both addiction and obesity are multifactorial and are thought to reflect an interplay of genetic and environmental factors.^10^ Although many argue that behavioral factors have a significant role in developing obesity in adulthood, it is well known that environmental insults at critical periods during fetal development can have life-long consequences for the health of the offspring. This process is known as fetal programming.^11^ There is extensive evidence that fetal priming can predispose the offspring to become obese^12, 13, 14^ and alter food choices.^15, 16, 17^ With respect to nutrition, epidemiological studies in humans support the conclusion that either undernutrition or overnutrition of the mother during pregnancy will have profound and long-term implications for the health of the offspring in adulthood, such as increased risk of developing adult obesity and the metabolic syndrome.^11, 18^

It is important to note that correlations between fetal programming and metabolic health of the offspring are stronger in human epidemiological studies of undernutrition than overnutrition because of the heavy confounding effects of prenatal and postnatal environment in the case of the latter. In the studies investigating the role of overnurtrition, exposure takes place throughout pregnancy and childhood; thus, the child is exposed and influenced by the food preferences of the mother, making it impossible to establish a specific link. With respect to addiction, evidence from human^1, 5, 19^ and animal studies suggests that gestational exposure to drugs of abuse leads to enhanced susceptibility for substance abuse in the offspring.^20^ However, to date, no human studies exist linking maternal consumption of junk food during pregnancy and predisposition to addiction for substance abuse.

We aimed in this study to elucidate whether maternal high-fat diet (HFD) can predispose the offspring that were not exposed to an obesogenic diet to hedonic behaviors to both drugs of abuse and highly palatable foods that might explain in part the growing rates of obesity that do not originate from a genetic predisposition. Furthermore, we intended to address whether these hedonic-like processes may share similar neurobiological mechanism to obesity.

## Materials and methods

### Subjects

C57BL/6N mice were used throughout the study. Female and male breeders were obtained from Charles River (Sulzfeld, Germany) and were housed under standard conditions (reversed light–dark cycle: lights on 1900 hours, temperature: 21±1 °C, humidity: 55±5%, food and water *ad libitum*) All procedures were approved by the Zurich Cantonal Veterinarian's Office.

### Maternal HFD exposure

**Figure 1:**
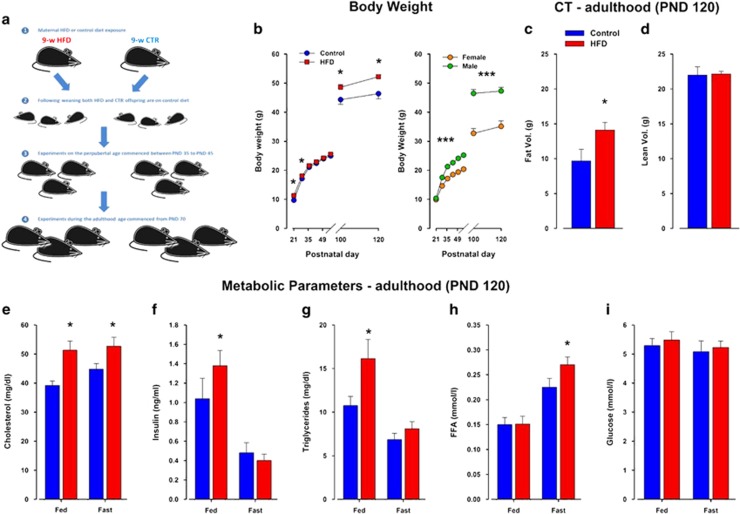
Offspring born to mouse dams exposed to maternal HFD develop a mild metabolic phenotype. (**a**) Schematic diagram illustrating the maternal HFD model employed. (**b**) Body weight development in HFD compared with control offspring. The scatter plot to the right show the sex differences between female and male offspring. Control *N*=92m and 52f and HFD *N*=64m and 43f. (**c**, **d**) Fat composition and distribution measured by CT-Scan in HFD and control offspring. (**e–i**) Metabolic parameters measured from blood samples taken from adult HFD and control offspring at PND 120. CT, computerized tomography; CTR, control; f, female; FFA, free fatty acid; HFD, high-fat diet; m, male; PND, postnatal day. *N*=9 m per group; *P<0.05, ***P<0.0001.

Female mice were exposed to HFD (Provimi Kliba, Kaiseraugst, Switzerland; containing 60% energy from fat) 3 weeks before mating, 3 weeks during gestation and 3 weeks during lactation (Figure 1a). Control dams were exposed to normal laboratory chow (Kliba 3430, Klibamühlen, Kaiseraugst, Switzerland).

### The effects of 9-week maternal HFD exposure on offspring weaned on normal chow

#### Hedonic phenotyping

Sucrose, alcohol and saccharin preferences: The tests were conducted in standard cages. Each cage was provided with two drinking tubes made from 15 ml polypropylene test tubes (Sarstedt, Germany). Offspring (sucrose test: *N*=16 (8m, 8f) per group, alcohol test: *N*=10 m per group, saccharin test: *N*=10 m per group) were first familiarized with drinking water from the two tubes and water consumption was measured every 24 h for 2 days (habituation phase). The preference test begun on the third day and lasted for 6 days for the sucrose and alcohol preference tests (that is, 2-day test phase for each of three ascending concentrations) and 4 days for the saccharin preference test. During the test, the mice were allowed free access to the two drinking tubes and food located on the cage top. The intake of the tested substance was calculated as an amount of consumed substance in mg per g body weight per 24 h.

Food preference: The food preference test was performed in metabolic cages (see Supplementary File). Offspring born to HFD-fed mothers (HFD offspring) and offspring born to chow-fed mothers (control offspring) were offered the choice between HFD and normal chow. *N*=6m and 6f per group. Mice were habituated to the apparatus (without HFD) for a time period of 2 days, and on day 3 the measures of food consumption was calculated. Mice were given a choice between HFD and normal laboratory chow and had *ad libitum* access to water. Chow and HFD intake was calculated as an amount of consumed HFD in mg per g body weight per 12 h.

Locomotor response to systemic amphetamine in the open field: The sensitivity to the psychotomimetic drug, amphetamine, was assessed measuring drug-induced locomotor activity in an open field apparatus and was monitored by the Ethovision tracking system (Noldus Technology, Wageningen, The Netherlands), which calculated a mobility score defined as the distance traveled per bin in successive 10-min bins (*N*=5 m per group).

Conditioned place preference test: The test apparatus consisted of two large chambers containing explicitly different visual and tactile cues (wall color and floor material) that were connected by a small shuttle chamber (neutral environment). Distance (per successive 5-min bins) and time spent in each compartment (in s) was monitored by a tracking system (Control *N*=24 (12m, 12f), HFD *N*=23 (11m, 12f); see Supplementary File).

Locomotor sensitization to cocaine: Sensitization to the locomotor-activating effects of 20 mg kg^−1^ of cocaine (intraperitoneal (i.p.) injection) was measured using the conditioned place preference test (CPP) apparatus. Mice (Control *N*=24 (12m, 12f), HFD *N*=23 (11m, 12f)) were injected i.p. with cocaine on days 1, 3, 5 and 7 (during the CPP test) and for cocaine sensitization on day 21. Basal locomotor activity was measured for 30 min, and thereafter locomotor activity to a cocaine injection was measured for 60 min.

Quantification of mRNA for deltaFosB: Control and HFD offspring (*N*=16 per group) were injected with 20 mg kg^−1^ cocaine for 5 days (1, 3, 5, 7, 21), following the last cocaine dose on day 21 when the brains were extracted 1 h post injection. Detailed methods for the qPCR are described in the Supplementary File.

#### Neurochemical and Neuroanatomical evaluations

Post-mortem neurochemistry: Levels of dopamine and its metabolites (dihydroxyphenylacetic acid; homovanillic acid) were determined using high-performance liquid chromatography according to procedures established before^21, 22^ and are described in detail in the Supplementary File.

Immunohistochemistry: Tissue processing and detailed methods of analysis are described in the Supplementary File.

#### PET Scan

The effect of an amphetamine challenge on the occupancy of available striatal D2R by dopamine was studied with positron emission tomography (PET). PET/CT scans were performed with an Argus (formerly VISTA eXplore) small-animal scanner (Sedecal, Madrid, Spain). Mice (*n*=6 for each group) were immobilized by isoflurane in air/oxygen (4–5% for induction and 2–3% to maintain anesthesia) and, 10 min before [^18^F]fallypride injection and scan start, *D*-amphetamine (2.5 mg kg^−1^ body weight) or an equivalent volume of saline (baseline) was injected i.p. (Supplementary File).

The effects of post-weaning HFD exposure: Following weaning, offspring born to control or HFD-exposed mothers (Control *N*=30 (16m and 14f), HFD *N*=28 (14m and 14f)) were exposed to either control chow or HFD until adulthood forming four groups: control offspring-chow, control offspring-HFD, HFD offspring-chow and HFD offspring-HFD. Body weight was monitored on a weekly basis, and the end of the experiment fat distribution was evaluated using CT-scan and metabolic blood parameters were taken.

Palatable food choice: Adult offspring born to control and HFD-exposed mothers (*N*=7 m per group) were given free choice to drink either water or a 1% sucrose solution and to eat normal laboratory chow or HFD in their home cage for a duration of 13 weeks. We have chosen the sucrose concentration of 1% as this concentration was used in the sucrose consumption test and showed increased preference over water by both the control and HFD offspring. The aim of our study was to investigate whether a synergistic effect will be induced when the animals were given a rather lower sucrose concentration in combination with HFD. Body weight was monitored on a weekly basis throughout the 13 weeks of palatable food choice exposure. The homeostasis model assessment of insulin resistance (HOMA-IR) was calculated using glucose and insulin concentrations obtained after 6 h of food withdrawal, using the following formula: fasting blood glucose (mg dl^−1^) × fasting insulin (μlU ml^−1^)/405.^23^ At the end of the experiment, fat distribution was evaluated using CT-scan, and metabolic blood parameter measurement and an insulin sensitivity test were performed (Supplementary File).

### Data analysis

Data were presented as mean±s.e.m. and analyzed employing the StatView statistical package (Abacuc, Baltimore, MD, USA). Two-way repeated measures analysis of variance (ANOVA) was used to analyze differences between control and HFD offspring, followed by Fisher's protected least significant difference test as warranted. Statistical significance was set at *P*-value of 0.05 or lower.

## Results

HFD offspring had an increased body weight shortly after birth compared with control offspring. An ANOVA revealed an effect of treatment (*F*1,19=4.67; *P*<0.05). Although there was no difference between the two groups until adulthood (Figure 1b), by postnatal day (PND) 120, HFD offspring kept on chow diet weighed more. These observations were supported by a significant main effect of PND (*F*3,57=404.16; *P*<0.0001) and a significant interaction of treatment × PND (*F*3,57=5.59; *P*<0.003). Subsequent *post hoc* test restricted to individual time periods showed that, although no significant differences were detected at PND 57 and 70 (*P*=0.91 and *P*=0.48, respectively), there was a significant difference in body weight at PND 100 and 120 (*P*<0.05 and *P*<0.01, respectively). Female offspring weighed significantly less than male offspring supported by a highly significant main effect of sex (*F*1,111=128.13; *P*<0.0001). At the end of the experiment, HFD offspring showed higher fat volume (*F*1,16=4.94; *P*<0.05) and slightly higher fat body mass only in the visceral depot (*F*1,16=6.04; *P*<0.03; Figure 1c), with no change in the lean body mass (Figure 1d). Furthermore, significant changes in circulating metabolic parameters such as cholesterol (*F*1,17=8.58; *P*<0.01), insulin (*F*1,17=43.69; *P*<0.0001), triglyceride (*F*1,18=44.10; *P*<0.0001) and free fatty acid levels were detected (*F*1,19=63.67; *P*<0.0001; Figure 1e–i).

### Altered hedonic behaviors to food in HFD offspring

**Figure 2:**
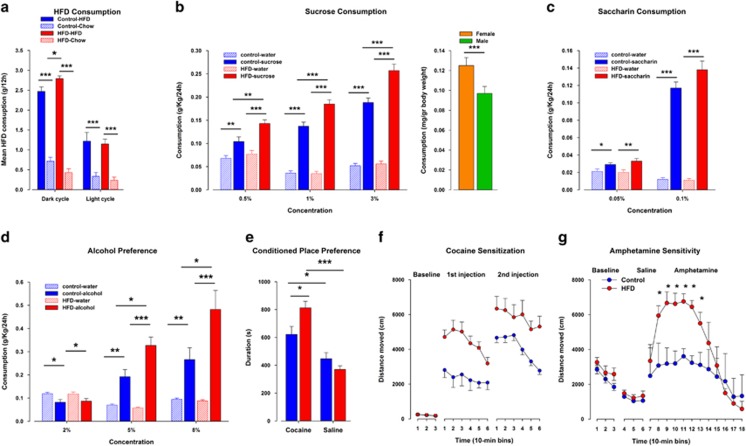
Offspring born to mouse dams exposed to maternal high-fat diet (HFD) show increased preference and consumption of palatable foods and drugs of abuse. (**a**) High-fat food preference test: mice from both groups were offered the choice between HFD and normal chow. *N*=(6m, 6f) per group. (**b**) Sucrose consumption test: in a free-choice protocol mice could choose either water or an ascending series of sucrose concentrations (1, 2 and 3%). The bar graph to the right show the sex differences between female and male offspring. *N*=16 (8m, 8f) per group. (**c**) Saccharine consumption test: animals were exposed to a free-choice protocol in which mice could choose either water or an ascending series of saccharine concentrations (0.5 and 1%). *N*=10 m per group. (**d**) Alcohol consumption test: animals were exposed to a free-choice protocol in which mice could choose either water or an ascending series of alcohol concentrations (2, 5 and 8%). *N*=10 m per group. (**e**) Conditioned place preference (CPP): measurement of conditioned place preference for cocaine in HFD compared with control offspring. *N*=48 per group. (**f**) Locomotor sensitization to cocaine: distance traveled on the first day of cocaine treatment and following 21 days of cocaine withdrawal in response to a 20 mg kg^−1^ cocaine challenge injection. *N*=17 per group. *N*=16 per group. (**g**) Locomotor reaction to systemic treatment with amphetamine: locomotor activity in the open field expressed as distance traveled (cm) per 10-min bin during the initial baseline phase, following saline administration and following a systemic injection of amphetamine (2.5 mg kg^−1^, intraperitoneal (i.p.)). *N*=5 m per group. *P<0.05; **P<0.001; ***P<0.0001. All values are means±s.e.m. f, female; m, male.

In the HFD preference test, an ANOVA revealed a highly significant effect of diet (*F*1,9=219.75; *P*<0.0001) and its interaction with treatment (*F*1,9=6.50; *P*<0.04), indicating a greater intake of high-fat food in HFD compared with control offspring. Both offspring groups hardly consumed the chow diet (Figure 2a). Furthermore, when offspring were given a free choice between drinking water and a sucrose solution, all the offspring preferred sucrose over water (*F*1,28=257.61; *P*<0.0001). An ANOVA yielded significant interactions of treatment × solution (*F*1,28=11.14; *P*<0.003) and a treatment × solution × concentration (*F*2,56=3.55; *P*<0.04), revealing that HFD offspring consumed significantly more from the sucrose solution at all concentrations tested as compared with controls, although there was no difference in water consumption between the groups (Figure 2b). Females drank significantly more liquid than males (*F1*,28=24.32; *P*<0.0001; Figure 2b). In contrast to the alterations in sucrose consumption, there was no difference detected in saccharin preference between the offspring groups. In addition, both offspring groups preferred the saccharin solution over water and consumed more from the saccharin solution in the highest concentration. These observations were supported by an ANOVA yielding significant main effects of solution (*F*1,17=183.42; *P*<0.0001) and concentration (*F*1,17=303.23; *P*<0.0001; Figure 2c).

### Altered hedonic behaviors to drugs of abuse in HFD offspring

When animals were given a free choice between water and alcohol, an ANOVA revealed a significant effect of treatment (*F*1,17=4.52; *P*<0.05) and its interaction treatment × solution × concentration (*F*2,34=5.82; *P*<0.006), indicating that the HFD offspring consumed more alcohol than their controls in the two highest concentrations (Figure 2d). Both offspring preferred the alcohol over water as indicated by a significant main effect of solution (*F*1,17=35.45; *P*<0.0001). In the CPP paradigm, before conditioning, offspring from both groups did not display any preference for one compartment over the other. In the preference test, females were significantly more active compared with male offspring. This observation was supported by a significant main effect of sex (*F*1,29=11.62; *P*<0.003; Figure 2e). In addition, both offspring groups displayed a preference to the cocaine-paired compartment relative to the saline-paired compartment supported by an ANOVA yielding a significant main effect of compartment (*F*1,29=33.51; *P*<0.0001). Furthermore, the HFD offspring expressed a significantly stronger preference to the cocaine-paired compartment as compared with the control offspring with no difference in their preference to the saline-paired compartment (Figure 2e) as was supported by a significant treatment × compartment interaction (*F*1,29=6.316; *P*<0.02). *Post hoc* comparisons yielded a significant difference in the paired compartment where HFD offspring spent significantly more time in the cocaine-paired compartment compared with control offspring (*P*<0.02). In the cocaine sensitization test (Figure 2f), no difference in baseline locomotor activity was detected between the offspring groups. HFD offspring displayed an increased reaction to the first as well as to the second cocaine injection compared with control offspring. This was supported by an ANOVA, indicating a significant effect of treatment (*F*1,39=18.00, *P*<0.0001), a significant effect of injection (*F*1,39=12.05, *P*<0.002) and a significant effect of time (*F*5,215=7.77, *P*<0.0001). The response to amphetamine is depicted in Figure 2g. Spontaneous locomotor activity and the response to the injection of saline were unaffected; however, the locomotor-enhancing effect of a systemic amphetamine administration was significantly enhanced in HFD offspring compared with their controls. An ANOVA yielded a significant effect of treatment (*F*1,6=6.00, *P*<0.05) and its interaction with 10-min bins (*F*11,66=5.30, *P*<0.0001).

### deltaFosB

**Figure 3:**
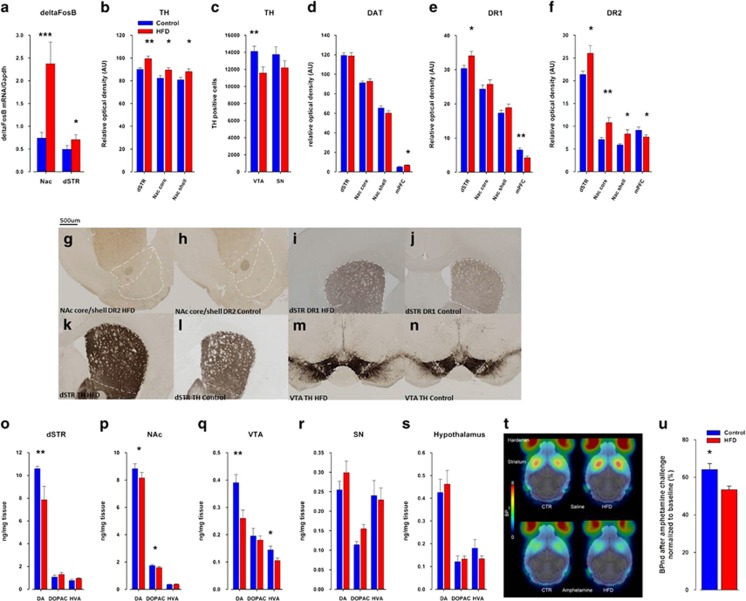
Maternal high-fat diet (HFD) exposure leads to alterations in the dopaminergic system. (**a**) mRNA expression of the transcription factor deltaFosB was significantly elevated in the nucleus accumbens (NAc) and dorsal striatum of HFD offspring compared with controls. Pregnant mice were exposed to HFD or normal laboratory chow (control) diet, and immunoreactivities of (**b**, **c**), tyrosine hydroxylase (TH), (**d**) dopamine transporter (DAT), (**e**) D1R and (**f**) dopamine receptors D2 (D2R) were assessed in the adult offspring (postnatal day 70, PND70). *N*=16 (8m, 8f) per group. (**g**–**n**) Representative images of coronal brain sections of adult (PND70) offspring derived from control and HFD-exposed mothers stained for D2R (**g**, **h**) in the NAC core and shell subregions and for D1R in the medial prefrontal cortex (mPFC) region (**i**, **j**) by immunohistochemistry. (**k**, **l**) Representative images of coronal brain sections for TH protein by immunohistochemistry in the dorsal striatum (dSTR; **k, l**) and the ventral tegmental area (VTA; **m, n**) of control and HFD offspring. (**o–s**) Levels of dopamine (DA) and its metabolites, 3,4-Dihydroxyphenylacetic acid (DOPAC) and homovanillic acid (HVA) were determined in post-mortem brain tissue using high-performance liquid chromatography (HPLC). Monoamine contents were measured in the dSTR, NAc, VTA, substantia nigra (SN) and hypothalamus. All monoamine levels are expressed as ng mg^−1^ fresh tissue weight. *N*=16 (8m, 8f) per group. (**t**) Positron emission tomography (PET) images (binding potentials, BP_nd_) superimposed on MRI templates (gray). Average PET images of three scans (with lowest tracer concentration) per group. (**u**) PET data (BP_nd_) normalized to the respective average accumulation under baseline conditions (%) to compare the relative response to amphetamine in both offspring groups. *N*=6f per group. *P<0.05; ** *P*<0.001; ***P<0.0001. All values are means±s.e.m. f, female; m, male.

Levels of deltaFosB were determined following a sensitization intermittent schedule of cocaine in control and HFD offspring. ANOVA revealed that levels of deltaFosB were significantly higher in HFD compared with control offspring and that the response was greater in the NAc (*F*1,25=14.55, *P*<0.0009) than in the dorsal striatum (dSTR; *F*1,25=5.48, *P*<0.03; Figure 3a).

### Neurochemical and neuroanatomical alterations in the brain reward system of HFD offspring

ANOVA revealed a significant effect of treatment indicating that HFD offspring displayed an increased elevation in the number of tyrosine hydroxylase (TH)-positive neurons in the NAc core (*F*1,20=10.40, *P*<0.005), NAc shell (*F*1,20=7.03, *P*<0.02) and dSTR (*F*1,20=24.15, *P*<0.0001; Figure 3b). Conversely, TH-positive neurons in the VTA were significantly reduced in HFD compared with control offspring (*F*1,20=15.45, *P*<0.0009; Figure 3c, m, n). Furthermore, maternal HFD exposure induced activation of the expression of dopamine receptor D2 (D2R) in the NAc core (*F*1,20=10.40; *P*<0.005) and shell (*F*1,20=6.45; *P*<0.02) subregions (Figure 3f–h) as well as in the dSTR (*F*1,20=6.74, *P*<0.02; Figure 3f), enhanced expression of D1R in the dSTR (*F*1,20=6.42, *P*<0.02; Figure 3e, Figure i, j) and increased expression of the dopamine transporter (Figure 3d) in the medial prefrontal cortex (*F*1,20=15.446, *P*<0.008; Figure 3d). To substantiate these findings we performed a post-mortem neurochemistry analysis that demonstrated that maternal HFD led in offspring to reduced levels of dopamine in the NAc (*F*1,48=4.69, *P*<0.05; Figure 3p), VTA (*F*1,42=9.03, *P*<0.005; Figure 3q) and dSTR (*F*1,42=4.42, *P*<0.05; Figure 3o), whereas no significant differences in dopamine levels were found in the substantia nigra (Figure 3r) and in the hypothalamus (Figure 3s).

### Striatal dopamine D2 receptor-binding availability using PET imaging

One-way ANOVA revealed that, under baseline conditions, [^18^F]fallypride accumulation and thus the number of available D2R were similar in the striatum of control and HFD offspring (control offspring 4.87±0.24, HFD offspring 5.16±0.31) (Figure 3t–u). Following the amphetamine challenge, a significant reduction of [^18^F]fallypride accumulation in the striatum in both groups (control offspring 3.48±0.13, HFD offspring 3.22±0.08) was observed. Moreover, after baseline normalization, [^18^F]fallypride accumulation was significantly lower in HFD than in control offspring, indicating a higher increase in synaptic amphetamine-induced dopamine release in the HFD compared with control offspring (*F*1,10=8.07, *P*<0.02).

### Post-weaning HFD exposure

**Figure 4:**
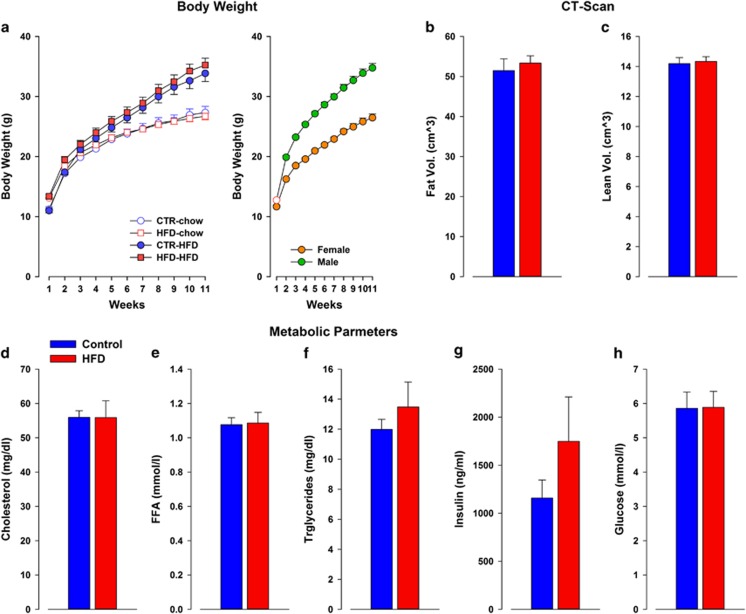
The effects of post-weaning HFD for 11 weeks on offspring born to either control or HFD-exposed mothers. (**a**) Body weights of control and HFD offspring were exposed to either chow or HFD diet for 11 weeks. Weights were weekly monitored. No difference was observed between control and HFD exposed to post-weaning chow diet. In addition, no difference in body weight was observed between control and HFD offspring exposed to HFD. The scatter plot to the right shows the sex differences between female and male offspring. Body composition of control and HFD offspring exposed to 11 weeks of post-weaning HFD. (**b**) No differences in fat mass and (**c**) lean mass utilized by CT-scan were detected. Post-weaning HFD did not lead to any difference in fasted plasma metabolic parameters between control and HFD offspring in any of the parameters measured: cholesterol (**d**), free fatty acid (FFA; **e**), triglycerides, (**f**) insulin (**g**) and glucose levels (**h**). Control *N*=30 (16m, 14f); HFD *N*=28 (14m, 14f). All values are means±s.e.m. CT, computerized tomography; f, female; HFD, high-fat diet; m, male.

No difference in body weight was observed between control and HFD exposed to post-weaning chow diet. In addition, no difference in body weight was observed between control and HFD offspring exposed to HFD. In addition, female offspring weighed significantly less than male offspring supported by a highly significant main effect of sex (*F*1,107=233.19; *P*<0.0001). ANOVA revealed a significant main effect of diet (F1,111=24.27; *P*<0.0001) and a significant interaction of diet × treatment (*F*10,1110=58.48; *P*<0.0001), which supported the fact that offspring exposed to post-weaning HFD weighed significantly more than offspring exposed to a post-weaning chow diet. The body weight of all animals during the 11 weeks was monitored as supported by a main effect of weeks (*F*10,1110=1134.77; *P*<0.0001; Figure 4a). The factor of sex and its interactions did not attain significance. Furthermore, post-weaning HFD exposure did not lead to any difference in fasted plasma metabolic parameters between control and HFD male offspring in any of the parameters measured: cholesterol (*F*1,13=0.07), free fatty acid (*F*1,13=0.02), triglycerides (*F*1,13=0.86) insulin (*F*1,13=1.54) and glucose levels (*F*1,13=0.002; Figure 4b–f). In addition, no differences were detected in the CT-scan of fat composition between control and HFD male offspring in any of the parameters measured: lean volume (*F*1,13=0.82), fat ratio (*F*1,13=0.28), fat volume (*F*1,13=0.26), subcutaneous fat (*F*1,13=0.17) and visceral fat (*F*1,13=0.41).

### The effects of exposure to a free-choice palatable diet during adulthood

**Figure 5:**
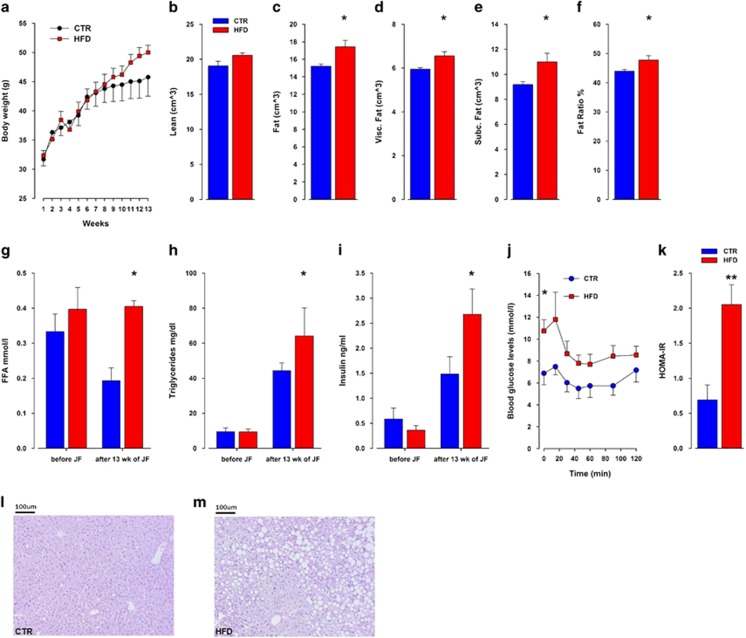
Food choice in offspring born to mouse dams exposed to high-fat diet (HFD) develop severe obesity and insulin resistance. (**a**). Control and HFD offspring during adulthood (postnatal day (PND) 70–160) were exposed to a choice paradigm; animals were given the choice in their home cage between control and HFD and between water to a 1% sucrose solution. (**b**–**f**) Fat composition and distribution as measured by computerized tomography (CT) scan. (**g**–**i**) Metabolic parameters measured from blood samples taken from HFD and control offspring at PND 160 (**j**). Insulin tolerance test (ITT) was performed in HFD and control offspring. Insulin (0.75  U kg^−1^ of body weight) was administered by intraperitoneal (i.p.) injection. Blood glucose levels were monitored at indicated times. (**k**) Plasma homeostasis model assessment of insulin resistance (HOMA-IR) was calculated by the fasting glucose and insulin levels. (**l**, **m**) Representative hematoxylin and eosin staining of liver sections of control (**l**) and HFD (**m**) offspring after a 13-week exposure to a choice diet. *N*=7 m per group; *P<0.05, **P<0.001. All values are means±s.e.m. m, male.

As shown in Figure 5a, an increase in weight gain was observed in HFD compared with control offspring following 13 weeks of free-choice palatable food exposure. ANOVA yielded a significant effect of weeks (*F*12,108=46.13; *P*<0.0001) and its interaction with treatment (*F*12,108=2.15; *P*<0.02). The increase in body mass was because of an increased fat pad weight as measured by CT scanning (Figure 5b–f). Whereas offspring did not differ in their lean mass (*P*=0.11), the HFD offspring displayed significantly higher mass of fat, % fat ratio, visceral fat and subcutaneous fat (*P*<0.02, *P*<0.04, *P*<0.02 and *P*<0.03, respectively) as compared with control offspring. Furthermore, the mice exhibited classical signs of worsened metabolic control, such as elevated free fatty acid, triglycerides and insulin levels (Figure 5g–i; *P*<0.05, *P*<0.003 and *P*<0.02, respectively). Maternal HFD exposure led to impaired insulin sensitivity in HFD offspring as measured by their response to an insulin challenge in the insulin tolerance test (Figure 5j). This observation was supported by ANOVA yielding a significant interaction of treatment × time point (*F*6,54=2.32; *P*<0.05). In addition, HOMA-IR was significantly increased in HFD compared with control offspring (*F*1,9=14.52; *P*<0.005; Figure 5k). The altered basal glucose levels observed in HFD offspring coupled to the increase in hepatic lipid accumulation observed in liver sections (Figure 5l) and HFD (Figure 5m) suggests a compromised metabolic regulation.

## Discussion

We demonstrated here that offspring born to HFD-exposed mothers compared with control offspring prefer at adulthood highly palatable foods and show enhanced sensitivity to and increased consumption of drugs of abuse. These behaviors were paralleled by relevant alterations of the dopaminergic system such as increased levels of the dopamine D1 and D2 receptors and decreased dopamine levels in the striatum. In addition, increased TH-positive neurons were detected in the striatum and reduced expression was found in the VTA. These neurochemical alterations likely underlie the behavioral changes related to higher sensitivity to drugs of abuse as well as to overconsumption of palatable foods. Contrary to offspring reared on normal laboratory chow or HFD post lactation, only when HFD offspring were given a free-choice palatable diet they developed obesity and the metabolic syndrome.

Maternal HFD-exposed offspring consumed more HFD and sucrose than control offspring. These results support findings of previous studies showing that maternal HFD can induce preference in rodents for palatable foods later in life.^15, 17, 24, 25^ No difference in preference to the artificial sweetener, saccharin, was observed between the offspring groups, suggesting that caloric value rather than taste drives the observed response. We observed that the offspring born to HFD-fed mothers only exhibited increased body weight shortly after birth and by PND 120 concomitant with a slightly higher fat body mass only in the visceral depot (Supplementary Fig. 6). This is not surprising as no changes in feeding behavior (Supplementary Table 1 and Supplementary Figs. 7) were observed. Similarly, a post-weaning HFD exposure did not exacerbate the mild metabolic phenotype observed, which is in line with previous reports.^26, 27^ Up until now studies have used HFD offspring, which were exclusively exposed to a single diet precluded from choice. In contrast, human daily food consumption is characterized by their ability to choose between consumption of palatable foods, generally calorie*-*dense and rich in sugar and/or fat, either in solid or liquid form to a healthy balance diet. In order to mimic the human daily consumption more closely, we gave the offspring at adulthood the possibility to freely choose between HFD and sucrose solution in addition to a normal laboratory chow diet and water. At the end of the study, HFD offspring developed obesity and various features of the metabolic syndrome. This indicates that the choice element of highly palatable foods may be important to induce hyperphagia in HFD offspring. Studies exposing adult rats to a free-choice diet showed overconsumption, increased abdominal fat and changes in glucose metabolism,^28, 29, 30, 31^ suggesting that the choice and the combination of high-energy food with sweet calorie-dense liquid in addition to the normal balanced chow and water leads to obesity. In contrast, studies using a non-choice HFD show only an initial increase in food intake as animals will overeat in the first few days, but after a short period of time will reduce their calorie intake to compensate for the body weight gain.^29, 32^ Recently, it was shown that a free-choice diet induced decrease in striatal D2R availability associated with increased caloric intake because of higher palatability and subsequently to overconsumption and adiposity.^28^ These results might explain why we did not observe any metabolic differences between control and HFD offspring reared on chow diet or on HFD (without a choice) and that changes emerged only following a free-choice palatable diet exposure. The data from the insulin tolerance test, which showed a higher initial starting glucose but a similar drop in relative levels, coupled to the increased HOMA-IR score and the increased lipid accumulation in the liver, suggest that HFD offspring have developed a mild systemic insulin resistance, which is most likely mediated by hepatic insulin resistance, although muscle insulin sensitivity is not yet affected. This could be due either to a specific phenotype of the employed model system, or to the length of the feeding paradigm.

In the current study we employed 1% sucrose concentration, which is commonly used in mice and rats^33, 34, 35^ and which is considered to be of intermediate palatability. The concentration was also chosen, as we could demonstrate in our animal model that even 1% of sucrose led to a significant increased preference over water, for both groups. Lastly, as higher concentrations of sucrose may have a significant impact on caloric intake because of the high-energy density and as the aim of the current study was to investigate whether a synergistic effect of diet and drink would lead to obesity, we chose to study the effect of 1% sucrose. Other studies in rats have used up to 30% of sucrose concentration,^29, 31^ which could possibly result in a different phenotypes than the one reported by us. Another limitation of the current study is that it was carried out in the home cages in the animal room, which precluded an exact food and/or liquid consumption measurement. To measure a precise caloric intake from food and/or liquid, the animals would have to be single-housed.^36^ As it is known that individual housing in mice is associated with a range of behavioral and physiological changes, such as increased food consumption,^37^ significant weight gain,^38^ increased sensitivity to stressor,^39^ elevated heart rate and core body temperature, these changes may have a potential to overshadow the results observed in our experiments.

The metabolic consequences on the offspring following different lengths of maternal HFD exposure have been characterized;^15, 24, 26, 40, 41^ however, conflicting results were reported with regard to the propensity of the adult offspring to develop obesity and metabolic disorders. Whereas some studies showed a mild obesity phenotype following maternal HFD exposure,^42, 43^ others reported protection from diet-induced obesity^27, 44^ or no effect at all.^26^ The discrepancies in the offspring's metabolic phenotype might be explained by the length of HFD exposure before conception. The maternal HFD exposure used in the present study did not induce any overt metabolic changes in the mother (Supplementary Figures 1–4) contrary to other studies using considerably longer exposure periods before conception.^15, 24, 26, 40, 41^ In the current study we studied the effect exclusively in obesity-prone C57BL6/N mice. Male mice exhibited significant weight gain in response to HFD even for a period of 3 weeks compared with control mice fed normal chow diet, a phenomenon that was not observed in female mice.

The role of the neural reward system in obesity has received increasing attention in recent years as both humans and rodents will consume palatable foods, that is, eat out of pleasure, in the absence of a metabolic need. Dopamine, the main neurotransmitter in the ventral striatum, which has a leading role in the brain reward circuitry and is associated with compulsive behaviors related to drug addiction,^45, 46^ is also released following ingestion of palatable foods.^8^ In line with the above, recent findings in humans using PET and functional magnetic resonance imaging have supported the idea that mechanisms of abnormal eating behaviors, including those observed in obese subjects, may have similarities to those underlying addiction to drugs of abuse.^47^ To address whether such a link exists in our model, we quantified the response to and consumptions of different drugs of abuse. We could show that when animals were given a choice between water and alcohol a significant increase in alcohol consumption was observed in HFD offspring. Similarly, HFD offspring showed enhanced cocaine-induced locomotor activity (Supplementary Figure 9) and a stronger cocaine-induced conditioned place preference compared with controls, indicating a greater degree of the reinforcing effect of the drug.^48^ In addition, HFD offspring showed enhanced response to the locomotor-activating effects of amphetamine as adults but not at the prepubertal age (Supplementary Figure 5), suggesting that maternal HFD exposure affects the development of the dopaminergic systems and that behavioral abnormalities only emerge in adulthood. Increased sensitivity to psychostimulant drugs, such as amphetamine and cocaine, reflects increased dopamine release in the NAc.^49^ Further, dysregulation of the dopaminergic system has been linked to the pathophysiology of many diseases such as drug addiction and hedonic overeating.^50^ Dopamine-mediated reward process in the striatum,^51^ such as the one involved in behavioral sensitization, requires a simultaneous stimulation of both D1 and D2 receptors. HFD offspring displayed increased expression of D1R in the dSTR and D2R in the NAC core and shell and in the dSTR. In keeping with the immunohistochemical results, decreased dopamine levels in the NAc, dSTR and the VTA of HFD offspring were detected. These results are similar to those reported in a study performed on metamphetamine users where cconcentration of the accumbal dopamine D1R was increased^52^ and low dopamine levels in both NAc and the striatum compared with control subjects were found.^53^ It was suggested that the increased dopamine D1R concentration could be part of a sensitization-type, positive feedback phenomenon occurring as a result of prolonged dopaminergic stimulation by the drug. Studies in rodents showed that supersensitivity of the dopamine D1R system explains some of the behavioral aspects of drug addiction, such as behavioral sensitization and drug self-administration.^54^ D1R is known to be the main postsynaptic target to mediate the action of amphetamine and cocaine in the NAc as D1R knockout mice show no effect to the locomotor-stimulating effect of cocaine.^55^ Rats sensitized to repeated cocaine injection also exhibit elevated D2R density.^56^ In a different neurodevelopmental model, prenatal stress produced increased accumabal D2R density concomitant with amphetamine-induced behavioral sensitization^57^ and showed increased vulnerability to develop amphetamine self-administration in the offspring.^58^ We observed decreased expression of TH-positive neurons, the rate-limiting enzyme of dopamine synthesis and a marker for dopamine neurons, in the VTA and increased expression in the NAc core and shell as well as in the dSTR. This could be explained by decreased enzymatic activity of TH in NAc and dSTR because of dephosphorylation of TH, which may result in reduced dopamine synthesis and therefore lower dopamine levels.^59, 60^ Similar results were reported in prenatal ethanol-exposed offspring exhibiting lower TH levels in the VTA^61^ associated with higher sensitivity to ethanol at adulthood.^62^ In addition, we could show that HFD offspring overexpressed deltaFosB in the NAc and in the dSTR. As DeltaFosB is a transcription factor implicated in long-term neuro-adaptations and is induced in the NAc following chronic exposure to drugs of abuse^63^ as well as to natural rewards,^64^ this could indicate that HFD offspring are more sensitive to the rewarding properties of drugs of abuse. In addition, mice overexpressing deltaFosB in dynorphin-positive accumbal medium spiny neurons show an increased motivation to obtain food reward because of reduced dopamine signaling.^65, 66^ Maternal HFD led to reduced dopamine levels in the striatum and in the VTA, and a compensatory increase in TH levels was detected in these regions. This may lead to increased transport of TH by neurofilaments from the VTA to the nerve terminals, resulting in lower TH levels in the VTA. Taking together, our results suggest an association between a striatal hypodopaminergic state of HFD offspring and their predisposition to increased consumption of highly palatable foods and higher sensitivity to drugs of abuse. Presynaptic dopamine release can be indirectly measured by assessing changes in binding of D2R radiotracers after pharmacological manipulation with dopamine-releasing agents measured by PET imaging,^67^ and [^18^F]fallyplide used in this study competes with endogenous dopamine for binding to D2Rs.^68, 69^ To the best of our knowledge, this work represents the first attempt employing a PET study to investigate the consequences of maternal HFD exposure. We observed a stronger reduction in striatal dopamine D2R radiotracers in the HFD offspring compared with controls, which can be interpreted as a measure of a greater increase of striatal dopamine release in response to the drug. A possible explanation for the discrepancies between our neurohistochemical results and the PET D2Rs findings may be due to the fact that for the immunohistochemical evaluations naive animals were used while the PET study was conducted in response to an amphetamine injection. The findings of this study mark an important attempt in advancing our understanding of the underlying mechanisms, leading to alterations in the reward system that result in increased hedonic behaviors following maternal HFD exposure. In addition, these results show more directly that excessive striatal dopamine transmission at the dopamine D2R level is involved in the augmented reaction toward drugs of abuse and imply for a hyper-responsive mesolimbic dopamine system. A PET study performed on human subjects reported that the striatal dopamine D2R level can predict the reinforcing effects of psychostimulant drugs.^70^ Subjects with high dopamine D2R level require a small dose of the drug to perceive a pleasant response, which is in agreement with our finding, meaning that HFD offspring may perceive the drug as more rewarding as control animals.

In summary, our findings demonstrate that maternal overnutrition during a period of 9 weeks leads to increased consumption of palatable foods and higher sensitivity to drugs of abuse in the offspring and that these hedonic behaviors may be mediated by the same neuronal pathways. We propose that this is due in part to hyporesponsive mesolimbic dopamine circuit, which is characterized by lower dopamine levels and enhanced striatal D1 and D2R expression. This may cause a higher vulnerability to addictive-like behaviors leading to an increased preference to consume palatable foods and sensitivity to drugs of abuse. On the basis of these results it will be important to determine long-term and developmental consequences of diets high in sugar and fat perinatally on limbic functions and motivated behaviors, as this may yield important new insights into the cause and treatment of compulsive eating.
