# Supplementary material for: Enhanced sensitivity to drugs of abuse and palatable foods following maternal overnutrition
Source: Transl Psychiatry. 2016 Oct 4;6(10):e911–. doi: 10.1038/tp.2016.176 (PMC5315546; doi:10.1038/tp.2016.176)
Supplement: Supplementary Information [file tp2016176x1.pdf]

SUPPLEMENTARY MATERIALS FOR

Enhanced sensitivity to drugs of abuse and palatable foods following maternal overnutrition

Dr. Daria Peleg-Raibstein[1]*, MSc. Gitalee Sarker[1], MSc. Kathrin Litwan[1], Dr. Stefanie D. Krämer[2], Prof. Simon M. Ametamey[2], Prof. Roger Schibli[2] and Prof. Christian Wolfrum[1]

[1]Laboratory of Translational Nutrition Biology, ETH Zurich, Schorenstrasse 16, 8603 Schwerzenbach, Switzerland

[2]Center for Radiopharmaceutical Sciences, ETH Zurich, Vladimir-Prelog-Weg 1-5/10, 8093 Zurich, Switzerland

*Correspondence: Dr. D Peleg-Raibstein, Laboratory of Translational Nutrition Biology, ETH Zurich, Schorenstrasse 16, 8603 Schwerzenbach, Switzerland. E-mail: daria-peleg@ethz.ch. Tel. 0041 44 655 73 50.

The file includes:

Materials and Methods

Results with statistics for all figures

Tables 1

Figures 10

Supplementary figure ledges

References

SUPPLEMENTARY MATERIALS AND METHODS

Phenotypic characterization of offspring

All offspring were weaned and sexed on postnatal day (PND) 21. Littermates of the same sex were caged separately. Offspring from 28 independent litters (14 HFD, 14 Control) were randomly selected for the behavioral, neurochemical, immunohistochemical, metabolic and PET tests. Both male and female offspring were included in most of the tests described below to assess potential sex-dependent effects [1] of maternal HFD exposure.

Maternal Behavior

Maternal behavior was measured in control and HFD dams in their home cage (5 dams per treatment group). Observations for each litter began on PND 1 until PND 20, 5 observations per day: 7.30 am, 12.30 am, 16.30 pm, 22.30 pm, 2.30 am (3 observations conducted during the dark phase and 2 observations conducted during the light phase). The observations were conducted using a video camera installed on the top of each cage (CMOS camera, 380 TVL, 3.6 mm, Conrad, Switzerland) connected to videorecorder (Sertek Modell SERT16-679Z, Sertek, Switzerland). A score of “0” or “1” depending whether each behavior category occurred or not. Each observation was scored for a duration of 5 min [2]. For the final analysis the frequency of each behavioral component was taken across 1 min bins (yielding 5 samples per observation). We have monitored 5 maternal categories which were described in detail elsewhere [3]. (1) *Carrying*: the dam grasps the pup by its skin and carries it to where the littermates are located. (2) *Licking*: the dams performs repetitive licking and head movements whilst hovering over the pups. (3) *Off nest*: (4) *Blanket nursing*: The dam is over the pups, relatively immobile, bilaterally symmetrical, with the head not depressed, and in a low dorsal arch posture. (5) *Arched back nursing*: The dam lies on its side or on its back with some of the pups attached to the nipples. The number of occurrences of each maternal behavior category was summed within 5-day blocks (Days 1-5, 6-10, 11-15 and 16-20) [4].

Behavioral Testing

The short- and long-term behavioral effects of maternal HFD exposure were assessed when offspring reached the prepubertal (PND 35-45) or the adult (PND 70-110) age. Prepubertal and adult stages of development were defined based on the completion of sexual maturity and behavioral manifestation [5]. Independent cohorts of animals were used for the behavioral experiments conducted during prepuberty and adulthood to avoid potential confounding transfer effects from behavioral testing. A naïve cohort of animals was used for neurochemical, neurohistochemical, molecular and PET evaluations. When the analyses did not yield a main effect of sex or significant interaction of sex

with maternal nutritional exposure the data of the two sexes were combined for the final analyses. All testing was conducted during the dark phase of the light-dark cycle.

Conditioned place preference (CPP)

The apparatus used to carry out the conditioned place preference (CPP) consisted of two large chambers containing explicitly different visual and tactile cues (wall color and floor material) that were connected by a small shuttle chamber (neutral environment). A digital camera mounted above the CPP apparatus captured images at a rate of 5 Hz and transmitted them to a personal computer running the Ethovision tracking system (Noldus Technology), which calculated a mobility score defined as the distance travelled per bin in successive 5 min bins. Additionally, the total time spent in the compartment was indicated in seconds. All mice underwent a pre-conditioning test (Pre Test) allowing them to freely explore the entire apparatus for one 15 min session. The measure for the Pre Test session was the duration in seconds spent in each compartment. Mice were then randomly assigned to saline/cocaine conditioning chambers for a total of eight conditioning sessions (cocaine was paired with the less preferred compartment). On each of the four saline conditioning days (Days 1, 3, 5, 7) animals received an intraperitoneal (i.p.) injection of 0.9% NaCl (saline) and were confined to the previously assigned saline-paired chamber for 30 min. For each of the four cocaine conditioning days (Days 2, 4, 6, 8) animals received an i.p. injection of cocaine hydrochloride at a dose of 20 mg/kg (Sigma–Aldrich, Switzerland) at a volume of 5 ml/kg dissolved in 0.9% NaCl− and were confined to the cocaine-paired chamber for 30 min. The preference test (Post Test) was performed 48 hours after the last conditioning day when mice were again permitted free access to all chambers for 30 min. The total time spent in each chamber during the Pre Test and Post Test was automatically recorded for subsequent statistical analyses.

Fat composition and distribution (CT-Scan)

Rodent computerized tomography allows to estimate the volumes of adipose tissue, bone, air, and the remainder, using differences in X-ray density, and distinguishes between visceral and subcutaneous adipose tissue. Measurements were performed non-invasively using a rodent computerized tomography (CT) scanner (LaTheta). Before CT scanning, animals were anesthetized using isoflurane (induction with 4-5% isoflurane in 600 cc O_2) and maintained under gas anesthesia (1.5-2.5 % isoflurane in 300 cc O_2) throughout the scan, which lasted about 5 min.

Energy expenditure and activity (Metabolic Cages)

Measurements of food and water intake, O_2 consumption/CO_2 production, were performed in an automatic feeding monitoring system coupled to an open-circuit indirect calorimetry system (TSE Phenomaster System). Mice were single housed under thermoneutral temperature conditions, food and water were available ad libitum and constantly monitored. Each cage was connected to a fresh air supply as well as the sample switch unit for drawing air samples from each cage. Cages (n=12)

were enclosed in a ventilated cabinet to precisely control ambient temperature and light. (control, f n=6; control, m n=6; HFD, f n=6, HFD, m n=6).

Insulin tolerance test (ITT)

Mice were food deprived overnight (12h-15h), but not water deprived. In the middle of the dark phase a 5 µL drop of tail blood was taken for baseline glycemia. Blood glucose was measured with a blood glucose monitor and the associated glucose test strips for glucose measurement (Accu-Check Aviva). Mice then received an i.p. injection of insulin (0.75 mU insulin/kg body weight, 10 µL of insulin solution/g body weight) and subsequent blood sampling (5 µL each time point) were collected at 15, 30, 60, 90 and 120 min post injection.

PET Scan

[^{18}F]fallypride was synthesized from its precursor tosyl-fallypride (ABX GmbH, Radeberg, Germany) according to [6]. Radiochemical yield was ≥ 20 % (decay corrected), radiochemical purity > 96 % and specific activity at the end of synthesis between 56 and 84 GBq/µmol. PET/CT scans were performed with an Argus (formerly VISTA eXplore) small-animal scanner (Sedecal, Madrid, Spain). Mice (n=6 for each group) were immobilized by isoflurane in air/oxygen (4-5 % for induction, 2-3 % to maintain anesthesia) and 10 min before [^{18}F]fallypride injection and scan start, *D*-amphetamine (2.5 mg *D*-amphetamine sulphate per kg body weight, 0.5 mg/mL in saline) or an equivalent volume of saline (baseline) was injected i.p. at time zero, 10 to 14 MBq (0.17 to 1.1 nmol) [^{18}F]fallypride was injected into a tail vein on the scanner bed and a dynamic PET scan of 60 min duration was started simultaneously. Body temperature was kept at 37°C and respiration rate around 60 per minute. A CT scan was performed at the end of the PET scan for anatomical orientation. PET data was reconstructed into twelve 1 min and twelve 4 min frames by a two-dimensional-ordered subset expectation maximization algorithm (2D-OSEM, 2 iterations, 16 subsets) with scatter and random but not attenuation correction. The voxel size of the reconstructed images was 0.3875 × 0.3875 × 0.775 mm^3.

PET quantitative analysis

PET data were analysed with the image analysis software PMOD (PMOD Inc., Zurich, Switzerland) and Microsoft excel. PET images were matched to the CT images and brain regions were delineated according to the mouse brain template implemented in PMOD. The volume of the combined striatum regions was 149 mm^3. Time-activity curves of brain regions were generated and binding potentials (BP_{ND}) were calculated by the Logan reference tissue analysis [7], where t*, the time to reach equilibration was 18 min and correction for k_2 (brain to blood rate constant) was neglected. A sphere in the cerebellum region of 52 mm^3 was used as the reference region [7]. Tracer concentrations in the reference region were calculated from the PET data and the specific radioactivity and scans with > 6.5 nM at 40 min after injection were excluded from the analysis, as receptor saturation with tracer was observed for these scans (data not shown). For each group, 6 scans were finally included in the

statistical analysis. PET images of the binding potentials (BP_{nd}) were generated with the PXMod module of PMOD. The resulting images of the three scans with lowest tracer concentration per group were averaged.

Immunohistochemistry

Adult (PND90) offspring were deeply anesthetized with an overdose of Nembutal (Abbott Laboratories) and perfused transcardially with 0.9% NaCl, followed by 4% phosphate-buffered paraformaldehyde solution containing 15% picric acid. The dissected brains were post fixed in the same fixative for 6 h and processed for antigen retrieval involving overnight incubation in citric acid buffer, pH 4.5, followed by a 90 s microwave treatment at 480. The brains were then cryoprotected using 30% sucrose in PBS, frozen with powdered dry ice, and stored at −80°C until further processing. Perfused brain samples were cut coronally at 30 µm thickness from frozen blocks with a sliding microtome. Six series of sections were collected, rinsed in PBS, and stored at −20°C in antifreeze solution until further processing. For immunohistochemical staining, the slices were rinsed three times for 10 min in PBS, and blocking was done in PBS, 0.3% Triton X-100, 5% normal serum for 1 h at room temperature. The following primary antibodies were used to study dopamine-related markers in various brain areas according to protocols established previously [8]: rabbit anti-TH (Santa Cruz Biotechnology; diluted 1:500), rat anti-dopamine D1 receptor (D1R) antibody (Sigma-aldrich; diluted 1:1000), rabbit anti-dopamine D2 receptor (D2R) antibody (AB5084P, Millipore Bioscience Research Reagents; diluted 1:500) and rat anti-dopamine transporter (DAT) antibody (MAB 369,Chemicon; diluted 1:3000). The tyrosine hydroxylase (TH) antibody (H-196) corresponds to 1-196 amino acids (with a deletion at 31-61) of human origin and the specificity of this antibody was previously reported [9].The D1R antibody (D2944) used recognizes 97 amino acids at the C-terminal end of the human D1R and its specificity was previously reported and validated [10, 11]. The D2R antibody was raised by immunizing rabbit against a peptide corresponding to 28 amino acid peptide sequence from the human D2R. The D2R antibody has previously been shown to be a specific and reliable marker to label D2Rs [10, 12, 13]. We confirmed it by using the dopamine D2R blocking peptide (AG221; Millipore Bioscience Research Reagents) which consists of 28 amino acid peptide sequence from the human D2R and is manufactured to block the staining of D2R antibody (AB5084P, Millipore Bioscience Research Reagents). Prior to immunostaining, the blocking peptide (50ug/ml) was mixed with D2R primary antibody (10ug/ml) in PBS containing 0.3% Triton X-100 and 2% normal goat serum. As control, only D2R primary antibody (10ug/ml) was added in PBS containing 0.3% Triton X-100 and 2% normal goat serum. Both solutions with or without blocking peptide were then incubated for 2 hours at room temperature. The staining was performed in two identical groups of brain sections, using the blocked antibody for one and the control for the other. Our result clearly demonstrates that the D2R staining observed in the medial prefrontal cortex (mPFC), dorsal striatum (DSTR) and nucleus accumbens (NAc) of the samples treated with the primary antibody (**Suppl Figure 10. a-c**) has been disappeared in the samples treated with the blocked antibody (**Suppl Figure 10. D-f**). The DAT antibody (MAB369) used recognizes N-terminus of human dopamine transporter fused to

Glutathione S-transferase and it is a common and reliable marker to label DAT [14]. All antibodies were diluted in PBS containing 0.3% Triton X-100 and 2% normal serum, and the sections were incubated free-floating overnight at room temperature. After three washes with PBS (10 min each), the sections were incubated for 1 h with the biotinylated secondary antibodies diluted 1:500 in PBS containing 2% NGS and 0.3% Triton X-100. Sections were washed again three times for 10 min in PBS and incubated with Vectastain kit (Vector Laboratories) diluted in PBS for 1 h. After three rinses in 0.1 m Tris-HCl, pH 7.4, the sections were stained with 1.25% 3,3-diaminobenzidine and 0.08% H_2O_2 for 10–15 min, rinsed again four times in PBS, dehydrated, and coverslipped with Eukitt (Kindler).

Stereological estimation of TH-positive dopaminergic midbrain neurons

The numbers of midbrain TH-positive dopamine neurons were quantified in the two relevant midbrain areas, namely in the ventral tegmental area (VTA) and substantia nigra (SN). For the latter region, TH-positive cells in both the substantia nigra pars compacta (SNc) and substantia nigra pars reticulata (SNr) were taken into account. The numbers of midbrain TH-positive dopamine neurons in either the left or right brain hemisphere were determined by unbiased stereological estimations using the optical fractionator method [15]. With the aid of the image analysis computer software Stereo Investigator (version 6.50.1; MicroBrightField), every section of a one-in-six series was measured, resulting in an average of six sections per brain sample. The following sampling parameters were used: (1) a fixed counting frame with a width of 40 µm and a length of 40 µm; and (2) a sampling grid size of 150 × 110 µm. The counting frames were placed randomly at the intersections of the grid within the outlined structure of interest by the software. The cells were counted following the unbiased sampling rule using the 40× oil lens [numerical aperture (NA), 1.3] and included in the measurement when they came into focus within the optical dissector [16].

Optical densitometry of dopaminergic markers in prefrontal cortical and striatal regions

Quantification of the immunoreactivity for TH, DAT, D1R, and D2R in striatal regions was achieved by means of optical densitometry using NIH ImageJ software. Optical densitometry was chosen because these dopaminergic markers are highly enriched at synaptic sites in the areas of interest. Digital images were acquired at a magnification of 2.5× (NA 0.075) using a digital camera (Axiocam MRc5; Zeiss) mounted on a Zeiss Axioplan microscope. Exposure times were set so that pixel brightness was never saturated. Pixel brightness was measured in the respective areas of one randomly selected brain hemisphere. In addition, pixel brightness was measured in the corpus callosum (for striatal measures) as background area. The background-corrected optical densities were averaged per brain region and animal. Four to six sections per animal were analyzed in the specimens. All immunohistochemical preparations were quantified in the dorsal striatum [caudate–putamen (CPu)], nucleus accumbens core (NAc core), and nucleus accumbens shell (NAc shell) (see below).

Delineation of brain regions

All brain areas of interest were delineated according to The Mouse Brain in Stereotaxic Coordinates by Franklin and Paxinos [17]. The following brain areas were included in the densitometric and stereological analyses: CPu (bregma +1.34 to +0.14 mm), NAc core and shell (bregma +1.60 to +0.98 mm), SN (bregma −2.80 to −3.64 mm), and VTA (bregma −2.92 to −3.64 mm). Schematic coronal brain sections of the brain areas of interest with reference to bregma are provided in corresponding figure legends.

Post-mortem neurochemistry

Levels of dopamine (DA) and its metabolites (dihydroxyphenylacetic acid, DOPAC; homovanillic acid, HVA) were determined using high performance liquid chromatography (HPLC) according to procedures established before [8, 18]. Animals assigned to the post-mortem neurochemical investigations were killed by decapitation on PND 70. HFD and control offspring were killed and dissected in random order. The brains were extracted from the skull within 1 min after decapitation and immediately frozen on dry ice and then were stored at -80 °C until dissection of the brain. For dissection, the frozen brain was placed ventral side up on an ice-chilled plate covered with filter paper and was cut with a razor blade into 1 mm thick coronal sections. The slices were placed on an ice cold dissection plate for the removal of discrete brain regions, using a 1 mm micropunch for the dorsal striatum (dSTR), medial prefrontal cortex (mPFC; including anterior cingulate and prelimbic cortices), nucleus accumbens (NAc; including core and shell subregions), substantia nigra (SN) and ventral tegmental area (VTA). Coronal sections were prepared along the following coordinates with respect to bregma: anterior-posterior +2.3 to+1.3, +1.3 to +0.3, –0.1 to –0.6, –1.2 to –2.2, and –2.8 to –3.8. Tissue punches from the left and right hemispheres of each brain area of interest were combined, weighed and placed in 1.5 ml polypropylene microcentrifuge tubes containing ice cold 300 µl 0.4M $HClO_4$ and homogenized using ultrasound. After centrifugation at 10,000×g for 20 min at 4 ˚C, the clear supernatant layers were removed into a 1 ml syringe and filtered through a 0.2 µm nylon filter to separate the insoluble residue. This solution was immediately frozen and stored at −80 ˚C until injection onto the HPLC system. For all brain regions an aliquot of 20 µl was injected in the HPLC system.

Chromatographic conditions for detection of monoamines and metabolites

A HPLC system coupled with an amperometric electrochemical detector (Decade II; Antec, Leyden, The Netherlands) was used to determine concentrations of DA, dihydroxyphenylacetic acid (DOPAC), and homovanillic acid (HVA). The samples were injected via a refrigerated autoinjector (ASI-100, Dionex, CA, USA) equipped with a 100 µl injection loop. The samples were separated on a reversed-phase column (125mm×3mm YMC column, Nucleosil 120-3 C 18, YMC Europe GmbH, Germany). An HPLC pump (P680, Dionex, CA, USA) connected to a pulse damper and a degasser was used to pump the mobile phase (see below) throughout the system. The working potential of the electrochemical glassy carbon flow cell (VT-03; Antec, Leyden, Netherlands) was +0.70V versus an ISAAC reference

electrode. A chromatography workstation (Chromeleon, Dionex, Olten, Switzerland) was used for data acquisition and calculations. The mobile phase consisted of 250 ml of HPLC-grade acetonitrile, 5l of aqueous solution containing 0.27mM sodium ethylendiammoniumtetraacetate ($C_{10}H_{14}N_2O_8Na_2 \bullet 2H_2O$), 0.43mM triethylamine ($C_6H_{15}N$), 8 mM potassium chloride, and 0.925 mM octanesulphonic acid ($C_8H_{17}O_3SNa$) which acted as an ion pairing reagent. The pH was adjusted to 2.95 by adding concentrated phosphoric acid. This was pumped through the system at a flow rate of 0.4 ml/min. The position and height of the peaks of the endogenous components were compared with samples of external calibrating standard solutions.

Quantification of mRNA for deltaFosB (real-time PCR)

For dissection, fresh brains from both offspring groups (*N* = 16 per group) were cut with a razor blade into 1 mm thick coronal sections. The slices were placed on an ice cold dissection plate for the removal of the dorsal striatum and nucleus accumbens using a 1 mm micropunch. Coronal sections were prepared along the coordinates mentioned above. For storage the tissue was frozen at −80°C. RNA from tissue was extracted using the Trizol-chloroform method (Invitrogen), treated with DNase (Ambion, Austin, Texas) for 30 min at 37°C, then converted to cDNA using High Capacity cDNA Reverse Transcription Kit [Applied Biosystems (ABI), Foster City, CA)]. 10 ng of cDNA was analyzed by real-time PCR using SYBR Green master mix (ABI) and the ΔΔCt relative comparison method, where target gene expression was normalized to expression of the housekeeping gene Gapdh. The forward primer of deltaFosB was 5’-AGGCAGAGCTGGAGTCGGAGAT-3’ and the reverse primer was 5’-GCCGAGGACTTGAACTTCACTCG-3’.

RESULTS WITH STATISTICS

Maternal Behavior

A 2 x 5 x 4 repeated measures ANOVA (treatment x observation sessions per day x 5-day blocks) was employed to assess differences in the 5 categories of maternal behavior.

Nursing behavior: The frequency of occurrence of this posture declined significantly through the lactation period ($F3,24 = 4.43$; $P < 0.02$). Control and HFD mothers showed similar changes in the total amount of nursing as evident by the lack of significant treatment x 5-day block interaction.

Licking/grooming: licking/grooming behavior generally decreased toward weaning ($F3,24 = 4.43$; $P < 0.02$) and was significantly more frequent in the light phase and licking/grooming occurred at the highest level on block Day 1-5 leading to a significant observations sessions per day x 5-day blocks interaction ($F12,96 = 2.03$; $P < 0.03$). There was no significant between control and HFD dams.

Carrying: Carrying decreased constantly across the lactation period as indicated by the significant main effect of 5-day block ($F_{3,24} = 3.27$; $P < 0.04$). No significant difference was detected between control and HFD mothers as was evident by a non-significant effect of treatment, either as a main effect or in interaction with 5-day block.

Mother off pups: out of nest behavior gradually increased during the lactation period leading to a significant main effect of 5-day block ($F_{3,27} = 4.03$; $P < 0.02$). Changes across days did not differ between control and HFD mothers. The main effect of treatment or its interaction with 5-day block did not attain statistical significance. Mothers off pups behavior was significantly more frequent in the dark phase, supported by a main effect of observations sessions per day ($F_{4,36} = 12.22$; $P < 0.0001$).

Passive nursing: passive nursing increased in the first 5-day block, Days 1-5, and then consistently declined as supported by a significant main effect of 5-day block ($F_{3,24} = 8.32$; $P < 0.0007$). There was no significant effect of treatment on passive nursing scores and there was no significant differences between the observation sessions per day.

Mothers' metabolic parameters

Mothers' body weight

Mothers' body weights prior to gestation and following 3 weeks of HFD exposure did not show any differences between mothers exposed to HFD and mothers exposed to the control diet ($F_{1,27} = 2.10$; P = n.s.; ***Supp Fig. 1a-d***).

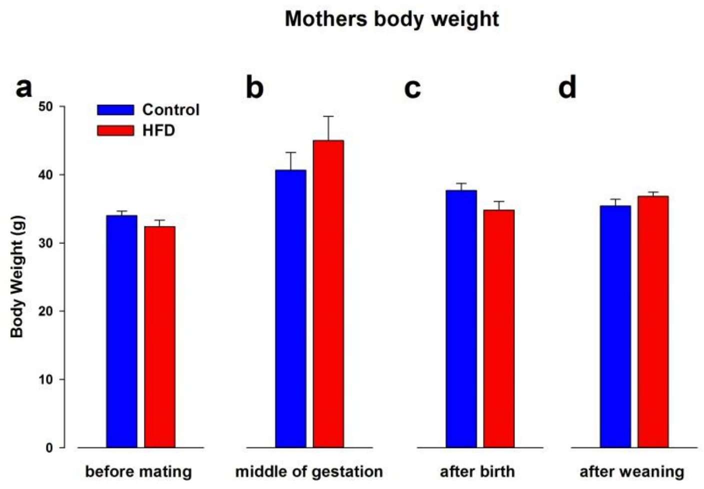

Supp Fig. 1.

Mothers' plasma metabolic parameters

Mothers' glucose levels prior to conception, after birth and after weaning did not yield any differences between mothers exposed to HFD and mothers exposed to the control diet (***Supp Fig. 2a-***

d). Similarly, no differences were detected between HFD and control dams in cholesterol, triglycerides, free fatty acids and insulin levels in blood after birth (***Supp Fig. 3a-e***).

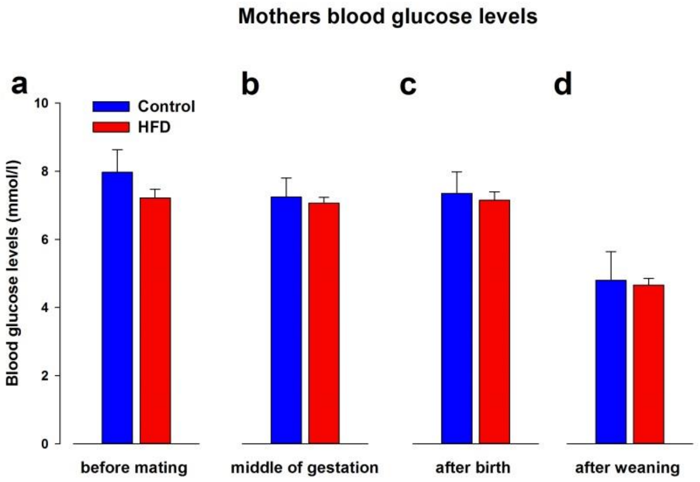

Supp Fig. 2.

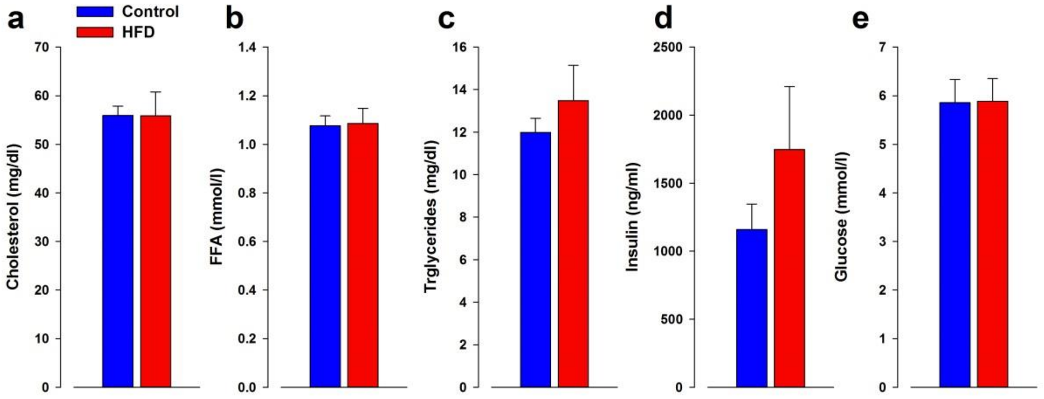

Supp Fig. 3.

Mothers' fat composition and distribution (CT-Scan)

In addition, mothers' CT-scan utilized after birth did not reveal any differences in the lean mass, total fat, %fat ratio, the subcutaneous and the visceral fat between the HFD and control dams (***Supp Fig. 4a-e***).

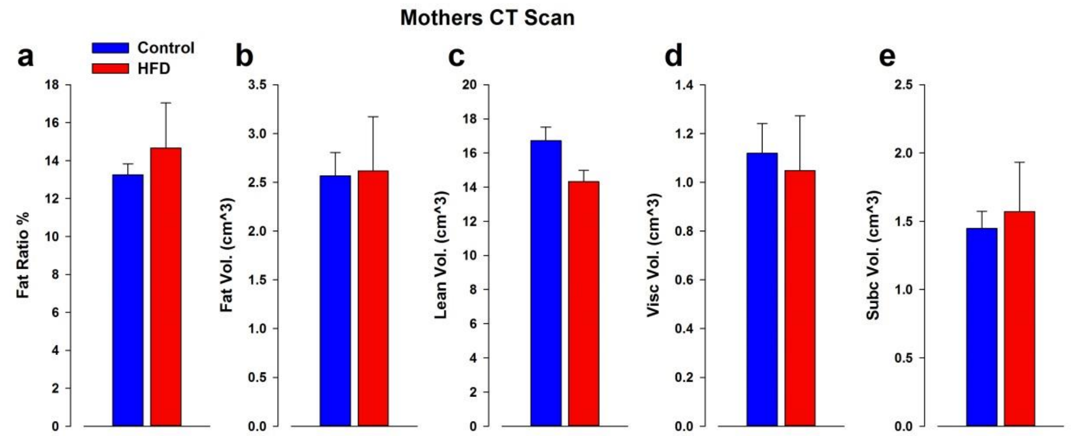

Supp Fig. 4.

Prepubertal (PND 35-45)

Amphetamine-induced locomotor activity

An ANOVA revealed no differences in locomotor activity between control and HFD offspring during the baseline and saline injection phases. Furthermore, following the amphetamine injection both offspring groups reacted in enhanced locomotor activity in response to the drug as indicated by a significant main effect of time (F11,66 = 19.46, $P < 0.0001$; ***Supp Fig. 5***).

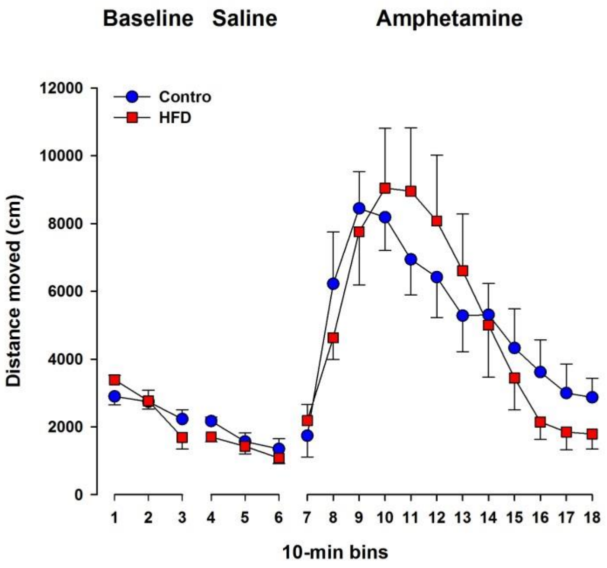

Supp Fig. 5.

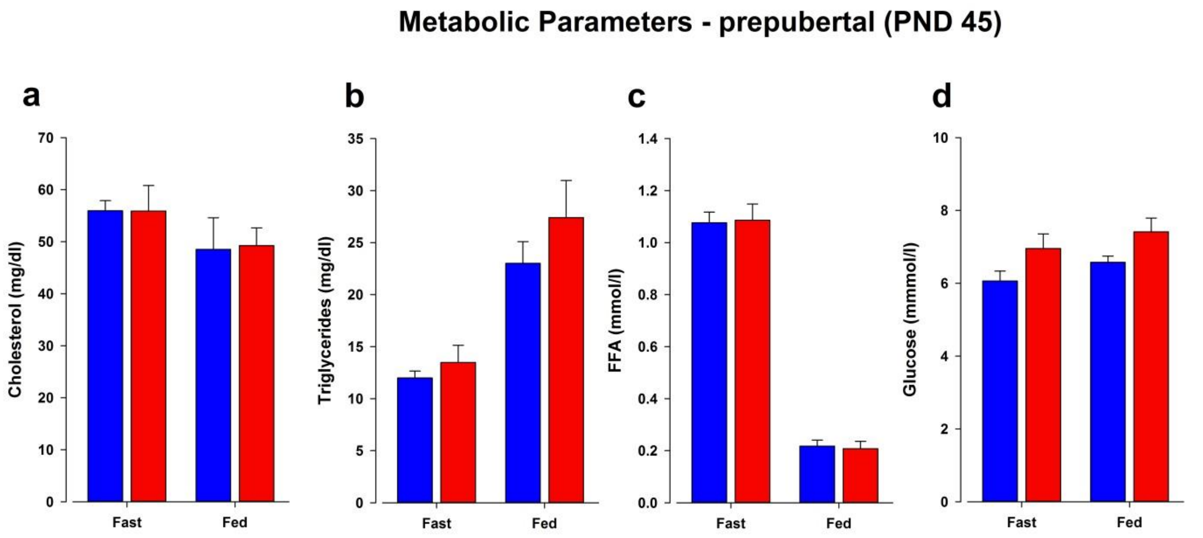

Supp Fig. 6.

Chow food intake (metabolic cages)

Both offspring born to HFD and control exposed mothers did not differ in consumption of chow diet during the light and dark cycle (*F*1,20 = 0.04). Animals ate more during the dark cycle than during the light cycle. This observation was supported by a 2 x 2 x 2 ANOVA (Treatment x Sex x Cycle) which revealed a significant main effect of Cycle (*F*1,20 = 159.86; *P* < 0.0001). There was no significant difference in food intake between female and male offspring (***Supp Fig. 7***).

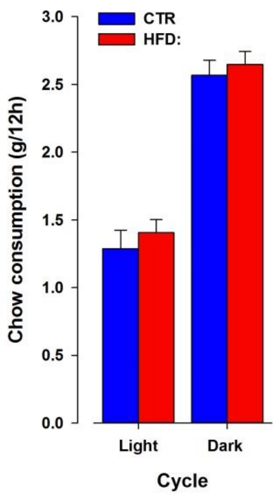

Supp Fig. 7.

	Food Intake (dark cycle)	Food Intake (light cycle)	VO2 (dark cycle)	VO2 (light cycle)	RER (dark cycle)	RER (light cycle)	Activity (dark cycle)	Activity (light cycle)
**Control**	2.57 ± 0.11	1.28 ± 0.14	3261.26 ± 208.88	3017.73 ± 190.03	0.79 ± 0.00	0.80 ± 0.01	37661.53 ± 3286.86	26432.22 ± 2261.00
**HFD**	2.65 ± 0.10	1.41 ± 0.10	3126.18 ± 88.18	2856.55 ± 76.65	0.80 ± 0.00	0.80 ± 0.00	41875.92 ± 4106.80	24128.36 ± 2152.64

Table 1.

Fat composition and distribution (CT-Scan) of offspring at PND 120

Maternal HFD treatment led to significantly higher fat ratio ($F1,16 = 5.71$; $P < 0.03$) (***Supp Fig. 8a***), and higher visceral fat ($F1,16 = 6.04$; $P < 0.03$) compared to control offspring (***Supp Fig. 8b***). There was a trend of a higher volume of subcutaneous adipose tissue in offspring born to HFD mothers fed a HFD ($F1,16 = 4.17$; $P = 0.058$) (***Supp Fig. 8c***).

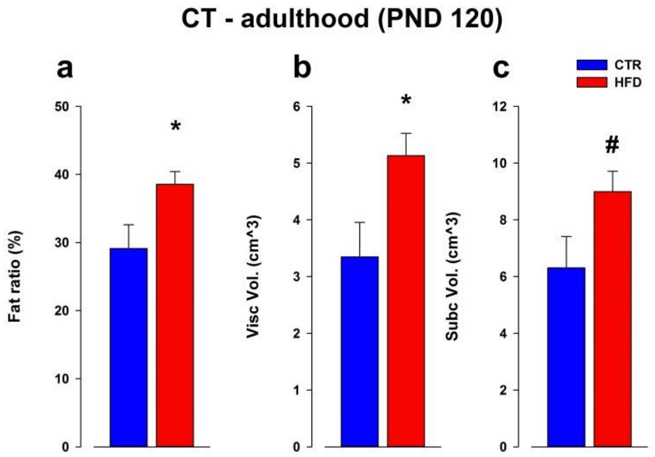

Supp Fig. 8.

CPP locomotor activity during the condition phase.

Four days of saline administration: A 2 x 2 x 2 x 4 x 6 repeated measures ANOVA (group x sex x drug x days x 5-min time bins) was conducted. Locomotor activity levels in response to the saline injection gradually increased across days in all offspring groups as was supported by a significant main effect of days ($F3,120 = 6.53$; $P < 0.0005$; **Supp Fig. 9a**). In addition, both offspring groups showed a gradual decline in locomotor activity levels from the first to the last 5-min block. This was supported by a highly significant main effect of 5-min time bins ($F5,200 = 51.38$; $P < 0.0001$). Female offspring displaced induced locomotor activity levels as compared to male offspring as was supported by a significant main effect of sex ($F1,40 = 18.03$; $P < 0.0001$; **Supp Fig. 9b**) and significant interactions of sex x days ($F3,120 = 5.10$; $P < 0.003$), sex x 5-min time bins ($F5,200 = 3.85$; $P < 0.003$) and sex x days x 5-min time bins ($F15,600 = 1.96$; $P < 0.02$). The factor of group and its interactions did not attain significance.

Four days of cocaine administration: A 2 x 2 x 2 x 4 x 6 repeated measures ANOVA (group x sex x drug x days x 5-min time bins) was conducted. Both offspring groups showed a gradual decline in locomotor activity levels from the first to the last 5-min block. This was supported by a highly significant main effect of 5-min time bins ($F5,200 = 15.26$; $P < 0.0001$). HFD offspring showed significantly higher locomotor activity levels in response to cocaine as compared to control offspring. This observation was supported by a significant main effect of group ($F1,40 = 15.90$; $P < 0.00004$; **Supp Fig. 9c**). In general the administration of a cocaine injection induced hyperlocomotor activity

compared to the saline injection in both control and HFD offspring as was supported by a significant main effect of drug ($F_{1,40} = 108.51$; $P < 0.0001$). Female offspring displaced induced locomotor activity levels as compared to male offspring as was supported by a significant sex x days interaction ($F_{3,120} = 5.10$; $P < 0.003$; **Supp Fig. 9d**) and a significant sex x 5-min time bins interaction ($F_{5,200} = 3.92$; $P < 0.003$).

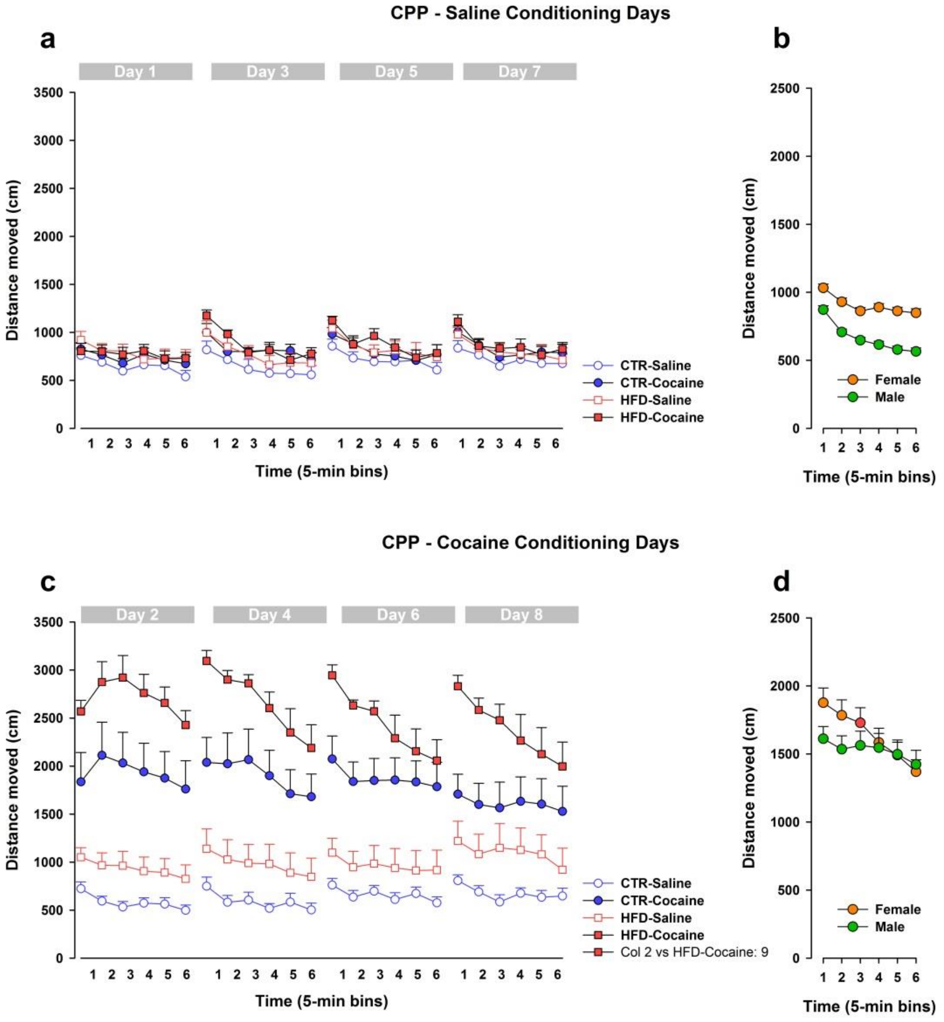

Supp Fig. 9

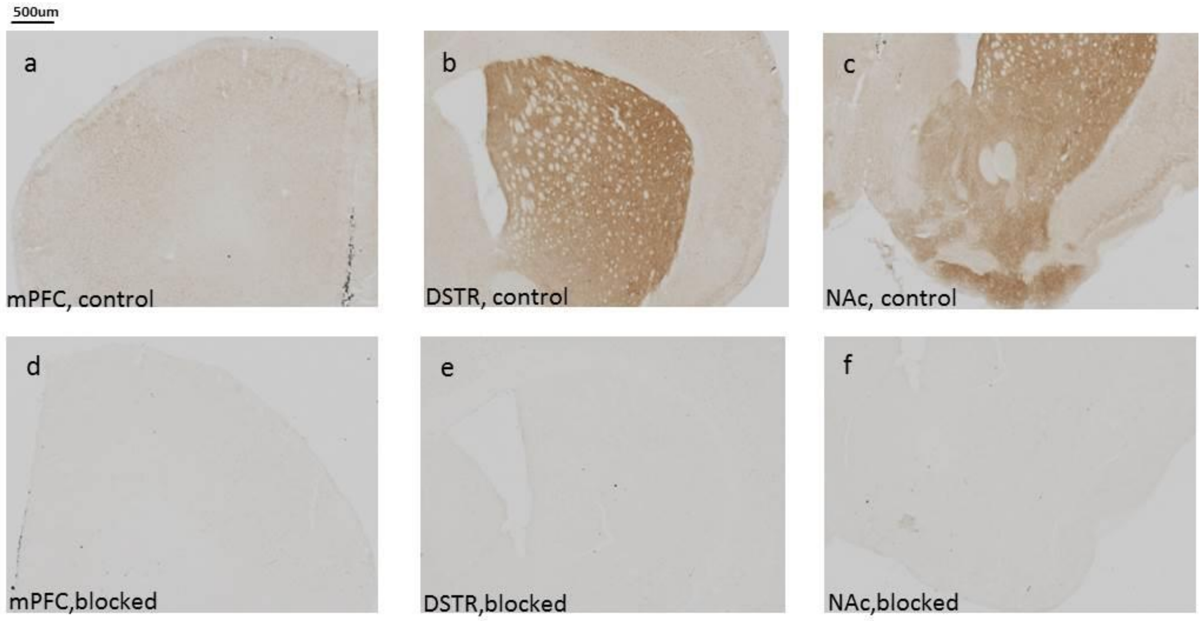
500um
a
mPFC, control
b
DSTR, control
c
NAc, control
d
mPFC,blocked
e
DSTR,blocked
f
NAc,blocked

Supp Fig. 10

SUPPLEMENTARY FIGURE LEGENDS

Supp Fig. 1

The body weights of dams exposed to either control or HFD diets were monitored before mating (**a**), during middle of gestation (**b**), after birth (**c**) and following weaning (**d**). No difference in body weight was detected between dams exposed to either control or HFD during the 4 different time periods.

Supp Fig. 2

Glucose levels of dams either exposed to control or HFD diets were monitored prior to conception (**a**), middle of gestation (**b**), after birth (**c**) and after weaning (**d**). There were no significant difference in glucose levels between mothers exposed to HFD and mothers exposed to the control diet.

Supp Fig. 3

Plasma metabolic parameters of dams exposed to either control or HFD diets were examined following weaning. No differences were detected between HFD and control dams in cholesterol (**a**), triglycerides (**b**), free fatty acids (FFA, **c**) and insulin blood levels (**d**) following birth.

Supp Fig. 4

CT-scan of dams exposed to either control or HFD diet utilized after birth did not reveal any differences in the lean mass (**a**), total fat, %fat ratio (**b**), the subcutaneous (**c**) and the visceral fat (**d**) between the HFD and control dams.

Supp Fig. 5

Locomotor reaction to systemic treatment with amphetamine: Locomotor activity in the open field expressed as distance traveled (cm) per 10-min bin during the initial baseline phase, following saline administration and following a systemic injection of amphetamine (2.5 mg/kg, i.p.).

Supp Fig. 6

Metabolic parameters measured from blood samples taken from HFD and control offspring at postnatal day 45. No significant changes in circulating metabolic parameters such as cholesterol, insulin, triglyceride and free fatty acid (FFA) levels were detected between control and HFD offspring (Fig. 7a-d).

Supp Fig. 7

The intake of normal laboratory chow of HFD and control offspring was monitored in the metabolic chambers. Both offspring born to HFD and control exposed mothers did not differ in consumption of chow diet during the light and dark cycle.

Table 1.

Energy balance monitored in the metabolic cages. Both offspring born to HFD and control exposed mothers did not differ in any of the parameters observed during the light and dark cycle: food intake, energy expenditure [VO2 consumption, respiratory exchange ratio (RER)] and physical activity.

Supp Fig. 8

Fat composition and distribution as measured with a computerized tomography (CT-Scan) displayed changes in fat disruption between HFD and control offspring. Maternal HFD treatment led to significantly higher fat ratio (***a***), higher visceral fat (**b**) compared to control offspring. Additionally, a trend was detected in subcutaneous adipose tissue in offspring born to HFD mothers compared to controls (***c***). *p < 0.05; #p = 0.06.

Supp Fig. 9.

Conditioned place preference (CPP) during each of the eight conditioning days. Offspring from both control and HFD groups were allocated to either saline or cocaine injections over a period of 8 days. **a.** Four saline conditioning days (days 1, day 3, day 5 and day 7). Offspring allocated to saline treatment received an injection of saline solution (0.9% NaCl). **b.** Female offspring showed enhanced locomtor activity levels compared to male offspring across the 4 days of saline injections. **c.** Four cocaine conditioning days (days 2, day 4, day 6 and day 8). Offspring allocated to cocaine treatment received a cocaine hydrochloride injection at a dose of 20 mg/kg and were confined to the cocaine-paired chamber for a period of 30 min. **d.** Female offspring showed enhanced locomtor activity levels compared to male offspring across the 4 days of cocaine injections.

Supp Fig. 10.

Dopamine receptor 2 (D2R) antibody specificity test. Representative images of coronal sections treated with the primary antibody (D2R) in the brain regions **(a)** medial prefrontal cortex (mPFC), **(b)** dorsal striatum and **(c)** nucleus accumbens (NAc). Representative images of coronal sections treated with the blocked antibody in the brain regions **(d)** medial prefrontal cortex (mPFC), **(e)** dorsal striatum and **(f)** nucleus accumbens (NAc).

REFERENCES

1. Clayton JA, Collins FS. Policy: NIH to balance sex in cell and animal studies. *Nature* 2014; **509**(7500)**:** 282-283.

2. Shoji H, Kato K. Maternal behavior of primiparous females in inbred strains of mice: a detailed descriptive analysis. *Physiol Behav* 2006; **89**(3)**:** 320-328.

3. Pryce CR, Bettschen D, Feldon J. Comparison of the effects of early handling and early deprivation on maternal care in the rat. *Dev Psychobiol* 2001; **38**(4)**:** 239-251.

4. Myers MM, Brunelli SA, Squire JM, Shindeldecker RD, Hofer MA. Maternal behavior of SHR rats and its relationship to offspring blood pressures. *Dev Psychobiol* 1989; **22**(1)**:** 29-53.

5. Spear LP. The adolescent brain and age-related behavioral manifestations. *Neurosci Biobehav Rev* 2000; **24**(4)**:** 417-463.

6. Mukherjee J, Yang ZY, Das MK, Brown T. Fluorinated benzamide neuroleptics--III. Development of (S)-N-[(1-allyl-2-pyrrolidinyl)methyl]-5-(3-[18F]fluoropropyl)-2, 3-dimethoxybenzamide as an improved dopamine D-2 receptor tracer. *Nucl Med Biol* 1995; **22**(3)**:** 283-296.

7. Muller Herde A, Keller C, Milicevic Sephton S, Mu L, Schibli R, Ametamey SM*, et al*. Quantitative positron emission tomography of mGluR5 in rat brain with [(18) F]PSS232 at minimal invasiveness and reduced model complexity. *J Neurochem* 2015; **133**(3)**:** 330-342.

8. Peleg-Raibstein D, Knuesel I, Feldon J. Amphetamine sensitization in rats as an animal model of schizophrenia. *Behav Brain Res* 2008; **191**(2)**:** 190-201.

9. Collo G, Bono F, Cavalleri L, Plebani L, Merlo Pich E, Millan MJ*, et al*. Pre-synaptic dopamine D(3) receptor mediates cocaine-induced structural plasticity in mesencephalic dopaminergic neurons via ERK and Akt pathways. *J Neurochem* 2012; **120**(5)**:** 765-778.

10. Romero-Fernandez W, Borroto-Escuela DO, Vargas-Barroso V, Narvaez M, Di Palma M, Agnati LF*, et al*. Dopamine D1 and D2 receptor immunoreactivities in the arcuate-median eminence complex and their link to the tubero-infundibular dopamine neurons. *Eur J Histochem* 2014; **58**(3)**:** 2400.

11. Mitrano DA, Pare JF, Smith Y, Weinshenker D. D1-dopamine and alpha1-adrenergic receptors co-localize in dendrites of the rat prefrontal cortex. *Neuroscience* 2014; **258:** 90-100.

12. Vuillermot S, Joodmardi E, Perlmann T, Ogren SO, Feldon J, Meyer U. Prenatal immune activation interacts with genetic Nurr1 deficiency in the development of attentional impairments. *J Neurosci* 2012; **32**(2)**:** 436-451.

13. Willi R, Weinmann O, Winter C, Klein J, Sohr R, Schnell L*, et al*. Constitutive genetic deletion of the growth regulator Nogo-A induces schizophrenia-related endophenotypes. *J Neurosci* 2010; **30**(2)**:** 556-567.

14. Meyer U, Nyffeler M, Yee BK, Knuesel I, Feldon J. Adult brain and behavioral pathological markers of prenatal immune challenge during early/middle and late fetal development in mice. *Brain Behav Immun* 2008; **22**(4)**:** 469-486.

15. Gundersen HJ, Bendtsen TF, Korbo L, Marcussen N, Moller A, Nielsen K*, et al*. Some new, simple and efficient stereological methods and their use in pathological research and diagnosis. *Apmis* 1988; **96**(5)**:** 379-394.

16. Howard CV, Reed MG. *Unbiased stereology: three-dimensional measurement in microscopy*, Second Edition edn. Garland Science/BIOS Scientific, New York, 2004.

17. Franklin KBJ, Paxinos G. *The mouse brain in stereotaxic coordinates* Elsevier Academic, Amsterdam, 2008.

18. Peleg-Raibstein D, Pezze MA, Ferger B, Zhang WN, Murphy CA, Feldon J*, et al*. Activation of dopaminergic neurotransmission in the medial prefrontal cortex by N-methyl-d-aspartate stimulation of the ventral hippocampus in rats. *Neuroscience* 2005; **132**(1)**:** 219-232.
