# Supplementary figures and images for: Enhanced sensitivity to drugs of abuse and palatable foods following maternal overnutrition
Source: Transl Psychiatry. 2016 Oct 4;6(10):e911–. doi: 10.1038/tp.2016.176 (PMC5315546; doi:10.1038/tp.2016.176)

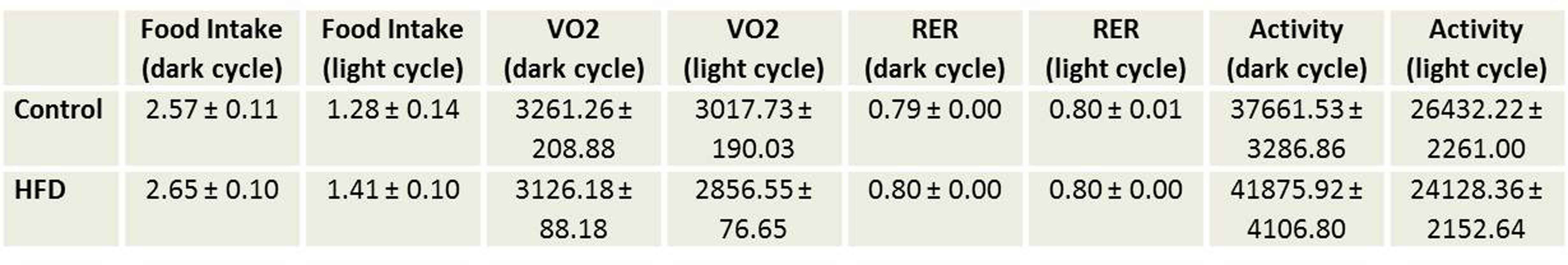

Supplement: Supplementary Table 1 [file tp2016176x2.tif]
